# Correction: Improvements in properties of polybenzoxazine-based laser-induced graphene (LIG) by alloying with polyimide and modeling of production process

**DOI:** 10.1039/d4na90069c

**Published:** 2024-06-20

**Authors:** Ibrahim Lawan, Panuwat Luengrojanakul, Krittapas Charoensuk, Hariharan Argunam, Cheol-Hee Ahn, Sarawut Rimdusit

**Affiliations:** a Center of Excellence in Polymeric Materials for Medical Practice Devices, Department of Chemical Engineering, Faculty of Engineering, Chulalongkorn University Bangkok 10330 Thailand Sarawut.R@chula.ac.th; b Polymer Engineering Laboratory, PSG Institute of Technology and Applied Research Neelambur Coimbatore 641062 India; c Department of Materials Science and Engineering, Seoul National University Seoul 08826 Korea

## Abstract

Correction for ‘Improvements in properties of polybenzoxazine-based laser-induced graphene (LIG) by alloying with polyimide and modeling of production process' by Ibrahim Lawan *et al.*, *Nanoscale Adv.*, 2024, **6**, 1556–1564, https://doi.org/10.1039/D3NA01026K.

The authors regret that the affiliations listed in the original manuscript were incomplete. The correct affiliations are as listed herein. In addition, the funding number affiliated to the National Research Council of Thailand (NRCT) fund had a typographical error. The correct funding number is N42A660910.

The Royal Society of Chemistry apologises for these errors and any consequent inconvenience to authors and readers.

